# Impact of Uterine Adenomyosis on Pregnancy Outcomes in Women Undergoing *In Vitro* Fertilization Treated With a Long-Term Pituitary Downregulation Protocol

**DOI:** 10.3389/fendo.2021.655803

**Published:** 2021-08-18

**Authors:** Jiaxin Zhang, Linli Hu, Zhiqin Bu, Yingpu Sun

**Affiliations:** Reproductive Medical Center, First Affiliated Hospital of Zhengzhou University, Zhengzhou, China

**Keywords:** adenomyosis, super long protocol, IVF, pregnancy outcome, endometriosis

## Abstract

**Background:**

Some studies have demonstrated that adenomyosis patients can achieve a comparable pregnancy outcome with women with normal uteruses, while there is no unanimous conclusion at present.

**Method:**

We recruited 65 adenomyosis patients and 260 frequency-matched control women with endometriosis at a ratio of 1:4 according to age. Clinical pregnancy rate, spontaneous abortion rate, and live birth rate were compared between these two groups after controlling other factors.

**Results:**

Compared with endometriosis patients, adenomyosis patients had a higher antral follicle count (AFC) (12.71 *vs* 11.65, *P*=0.027). Though the two groups had the same number of embryos transferred, adenomyosis patients had an obviously declined implantation rate (31.91% *vs* 46.74%, *P*=0.005), clinical pregnancy rate (47.06% *vs* 64.42%, P=0.028), live birth rate (31.37% *vs* 54.81%, P=0.004), and significantly increased spontaneous abortion rate (33.33% *vs* 13.43%, P=0.034). Multivariate logistic regression analysis showed that adenomyosis had adverse influences on pregnancy outcome when age and the number of embryo transfers were controlled (adjusted OR=0.361, P=0.003).

**Conclusion:**

Even after being matched with age, adenomyosis still had adverse influences on the pregnancy outcome of IVF in patients undergoing the long protocol.

## Introduction

Adenomyosis, which can influence uterine peristalsis, the transportation of sperm in the uterine cavity, and embryo implantation, finally leads to fertility decline (1, 2). Assisted reproductive technologies (ART) are currently good methods for solving infertility issues (3). Moreover, *in vitro* fertilization and embryo transfer (IVF-ET) has become a common method to help infertility caused by adenomyosis (4). However, studies have indicated that these patients with adenomyosis have a declined tolerance to embryos and decreased maintenance ability of pregnancy, which leads to a lower IVF success rate compared with controls (5).

Gonadotropin-releasing hormone analogue (GnRH-a) is a synthetic decapeptide compound which can downregulate pituitary function causing a temporary low estrogen state (6). Since adenomyosis is an estrogen-dependent disease, the use of long-acting GnRH-a can control the growth of endometriosis by inhibiting ovary function (7). In addition, in the process of IVF, the pretreatment of long-acting GnRH-a can not only effectively inhibit LH surge, but can also shrink uterus shape, which is beneficial to pregnancy outcome improvement (8, 9). Some scholars have found that, in adenomyosis patients who received a super long protocol (pretreatment of long-acting GnRH-a for ≥3 months) in IVF treatment, the pregnancy outcome was comparable to that of the controls with a normal uterus (10). Currently, the use of long-acting GnRH-a for several cycles before conducting IVF is widely adopted at many centers for adenomyosis patients.

However, after pregnancy the estrogen level is higher than physiological status. Elevated estrogen level will lead to the recovery of the shrunken uterus, which will eventually increase the spontaneous abortion rate (11). Therefore, the spontaneous abortion rate should be fully taken into account and the live birth rate should be seen as the primary endpoint in such studies (12). In addition, the pregnancy outcome of IVF is also influenced by other factors, including age, number of transferred embryos, and quality of embryos, etc. Thus, these factors concerning the influences of adenomyosis on pregnancy outcome should not be ignored in studies.

Therefore, in order to figure out the influences of adenomyosis on pregnancy outcome undergoing long IVF protocol treatment, we carried out this matched case-control study under the preconditions of fully taking into account age and other pregnancy associated factors, and only considering the results regarding the first cycle.

## Materials and Methods

This study has been approved by the Institutional Review Board (IRB) of First Affiliated Hospital of Zhengzhou University in January 2020. All patients undergoing IVF treatment at our center have agreed to us using their medical record data for research and written informed consent was obtained. The data were selected from our electronic database containing detailed information for IVF from January 2016 to July 2019. For adenomyosis patients, those combined with endometrioma, or with the history of endometrioma excision were excluded. The control group consisted of patients who had endometriosis proven by laparoscopic surgery or endometrioma confirmed by ultrasound. In order to avert the influences of age on pregnancy outcome, adenomyosis patients and endometriosis patients were matched by age at a ratio of 1:4. Only patients in their first IVF cycle were included. All patients in both groups were pretreated by long-acting GnRH-a treatment for 1-6 months before ovarian stimulation.

The diagnostic criteria of adenomyosis were as follows: (1) The clinical manifestations of secondary progressive dysmenorrhea, menorrhagia, or menostaxis; (2) pelvic examination indicating that the uterus was equally enlarged and had a hard texture and pressing pains; and (3) the presence of three or more sonographic criteria: asymmetrical thickening, cysts, hyperechoic islands, fan-shaped shadowing, echogenic subendometrial lines and buds, translesional vascularity, an irregular junctional zone, and interrupted junctional zone (13, 14). All patients with adenomyosis received repeated ultrasound examinations with the same ultrasound machine model by two professional doctors in our center. All three criteria mentioned had to be met for patients to be diagnosed with adenomyosis.

All patients received long-acting triptorelin, 3.75 mg i.m. every 28 days for 1-6 cycles before the start of IVF treatment. Around 30-45 days after the last injection of GnRH-a, recombinant FSH was used to stimulate follicle growth at the dose of 100-300 IU. Four days after the use of FSH, gonadotrophin dosage was adjusted according to the follicular development and hormone level. When the diameter of at least two leading follicles reached 18 mm, human chorionic gonadotrophin (hCG) was used to trigger ovulation. Thirty-seven hours later, oocyte retrieval was performed.

Routine IVF or intra-cytoplasmic sperm injection (ICSI) was performed within 4-6 h after oocyte retrieval according to the condition of sperm collected from the husband. Three days after oocyte retrieval, the quality of embryos was assessed by an embryology expert according to the cell numbers, the quantity of fragments, and whether multinucleated blastomeres existed. Only patients transferred with high quality embryos (≥ six blastomeric cells, the quantity of fragments ≤ 20%, and no multinucleated blastomeres) were included.

The luteal support protocol was started by progesterone injection on the day of oocyte retrieval at the dosage of 40 mg i.m. bid. On embryo transfer day, progesterone was adjusted to 60 mg i.m. qd, and 10 mg po bid of dydrogesterone tablets was added. Serum hCG level was examined 14 days after transfer. Biochemical pregnancy was diagnosed with hCG ≥5 IU/L. Five weeks after transfer, if the gestational sac and heart beat were seen inside the uterus, the patient was diagnosed with clinical pregnancy. The well-trained staff in our center performed follow-up for all patients receiving IVF. The follow-up of those with clinical pregnancy will last until delivery.

Statistical analysis was performed by SPSS17.0. Measurable data were expressed by mean ± standard deviation. Two independent T tests were adopted to compare the difference in two groups for measurable data. The Chi-square test was used for category data. Multivariate logistic regression analysis was used to analyze the relation between adenomyosis and other clinical variables and live birth rate. P<0.05 was considered statistically significant.

## Results

The study recruited 65 adenomyosis patients in total. Overall, 260 endometriosis patients were matched with adenomyosis patients at a ratio of 1:4 according to age. They had a similar rate of infertility reasons, diagnosis, and cancelled causes. Finally, the two groups respectively included 51 adenomyosis patients and 208 endometriosis patients who received embryo transfer (**Table 1**).

**Table 1:** Summary of basic information for all patients included in this study.

	Adenomyosis	Endometriosis
No. of patients included	65	260
Primary infertility (%)	39 (60.00%)	163 (62.69%)
Secondary infertility (%)	26 (40.00%)	97 (37.31%)
Diagnosis		
* Only endometriosis*	–	116 (44.62%)
* Only adenomyosis*	18 (27.69%)	–
* Combined with tubal factor*	18 (27.69%)	89(34.23%)
* Combined with anovulatory*	5 (7.69%)	12 (4.62%)
* Combined with male factor*	4 (6.15%)	15 (5.77%)
No. of patients with cancelled ET	14	52
* No oocyte retrieved*	1	5
* No embryos available*	2	9
* OHSS*	7	23
* P elevation on HCG day*	0	2
* Thin endometrial thickness*	0	0
* Uterine cavity fluid*	1	1
* Others*	3	12
No. of patients undergoing ET	51	208

ET, embryo transfer; OHSS, ovarian hyper-stimulation syndrome; P, progesterone level; HCG, human chorionic gonadotrophin.

There were no differences between the two groups in regard to age, infertility duration, body mass index (BMI), basal follicle stimulation hormone (FSH), and number of oocytes retrieved. Except that adenomyosis patients had an increased antral follicle count (AFC) (12.71 ± 6.43 *vs* 11.65 ± 5.50, *P*=0.027), there was no difference between the two groups on the length of Gn duration (**Table 2**).

**Table 2:** Basic parameters and clinical outcome for patients undergoing IVF/ICSI with and without uterine adenomyosis.

	Adenomyosis	Endometriosis	*P* value
	*N* = 65	*N* = 260	
Age (year)	32.25 ± 3.76	32.15 ± 3.69	0.735
Infertility duration (year)	3.75 ± 2.75	4.07 ± 2.98	0.989
BMI (kg/m^2^)	23.50 ± 3.70	21.99 ± 3.12	0.116
Basal FSH (mIU/mL)	5.94 ± 1.72	6.98 ± 2.43	0.131
AFC	12.71 ± 6.43	11.65 ± 5.50	0.027
Gn duration (day)	13.75 ± 2.77	13.39 ± 2.76	0.968
No. of oocytes retrieved	11.15 ± 6.37	11.92 ± 6.37	0.910

Data were mean ± standard deviation unless otherwise noted;

IVF, in vitro fertilization; ICSI, intracytoplasmic sperm injection; BMI, body mass index; FSH, follicle stimulation hormone; AFC, antral follicle count; Gn, gonadotrophin; IU, international unit.

Though the two groups had the same number and a similar quality of embryos transferred, the clinical pregnancy rate of adenomyosis patients was lower (47.06% *vs* 64.42%, P=0.028). In addition, they had a significantly increased spontaneous abortion rate (33.33% *vs* 13.43%, P=0.034), obviously declined implantation rate (31.91% *vs* 46.74%, *P*=0.005), and live birth rate (31.37% *vs* 54.81%, P=0.004) when compared with patients with endometriosis (**Table 3**).

**Table 3:** Pregnancy outcome for patients undergoing IVF/ICSI with and without uterine adenomyosis.

	Adenomyosis	Endometriosis	*P* value
	*N* = 51	*N* = 208	
No. of embryos transferred	1.84 ± 0.37	1.84 ± 0.37	0.950
Implantation rate (%)	31.91% (30/94)	46.74% (179/383)	0.005
Clinical pregnancy rate (%)	47.06% (24/51)	64.42% (134/208)	0.028
Spontaneous abortion rate (%)	33.33% (8/24)	13.43% (18/134)	0.034
Live birth rate (%)	31.37% (16/51)	54.81% (114/208)	0.004

Data were mean ± standard deviation unless otherwise noted.

Multivariate logistic regression analysis showed that even in long-protocol IVF cycles, adenomyosis had adverse influences on pregnancy outcome when age and the number of embryos transferred were controlled (adjusted OR=0.361, 95%CI: 0.185-0.705, *P*=0.003) (**Table 4**).

**Table 4:** Factors associated with live birth rate by multivariate logistic regression analysis.

	OR	95% CI	*P* value
Age (y)	0.901	0.839-0.968	0.004
No. of embryos transferred	4.438	2.790-7.058	0.000
Adenomyosis			
No (With endometriosis)	1.000 (Reference)		
Yes	0.361	0.185-0.705	0.003

OR, odds ratio; CI, confidence interval.

## Discussion

For adenomyosis patients undergoing IVF treatment, the oversize of the uterus, tissue necrosis factors, and other toxicants owing to chronic inflammation caused by invading endometrial glands, all have adverse influences on embryo implantation (15). Therefore, most studies showed that adenomyosis can reduce the embryo implantation rate and clinical pregnancy rate and increases the abortion rate in IVF. Currently, long-acting GnRH-a has been widely applied in endometriosis. The long protocol of using long-acting GnRH-a can not only effectively control the growth of the ectopic endometrium but can also reduce the detrimental effects of cytotoxic cytokines and oxidative stress, obviously improving the pregnancy outcome of endometriosis patients (16). When treated with long-protocol IVF, some studies have shown that adenomyosis patients can achieve the same pregnancy outcome as that of women with normal uteruses (4, 17). However, contrary to the results of the meta-analysis, the long protocol did not affect the clinical pregnancy rate in this article. Reasons for the controversy are probably as follows: there were two retrospective cohort studies included in long-term protocol group, Mijatovic chose the IVF protocol long-term (≥3months) GnRH agonist to downregulate pituitary function (10); Costello chose E2<120 Pmol/L, what is more they pretreated patients with the oral contraceptive pill (18). While in our study the patients received long-acting triptorelin for 1-6 cycles, most of whom were treated for two cycles. Different treatments may underline different results.

There are some challenges to studying endometriosis and adenomyosis. First, the diagnosis criteria of adenomyosis are not uniform. Moreover, because endometriosis and adenomyosis usually coexist, most studies have not distinguished the two diseases (19); second, there are many other factors influencing pregnancy outcome in the process of IVF. Some studies do not control age, quality of embryos, and other pregnancy-related factors (20). In addition, live birth rate and spontaneous abortion rate are not well evaluated.

In the current study, all recruited adenomyosis patients were diagnosed by the experienced clinical doctors in our center, and adenomyosis patients with endometrioma and patients with a history of endometrioma excision were excluded. More importantly, patients in both groups received the same IVF treatment using the long protocol. The live birth rate was compared when age and number of embryos were controlled in the logistic progression.

It is known that the quality of embryos and endometrial receptivity are seen as two important factors influencing pregnancy outcome. Our study showed that compared with the control group, adenomyosis patients had a similar number of available embryos and high-quality embryos, but a significantly lower implantation rate and clinical pregnancy rate. Therefore, adenomyosis may not influence the quality of embryos but has adverse influences on endometrial receptivity which have also been proved by another relevant study (21).

In addition, our study showed that compared with endometriosis patients, the clinical pregnancy rate of adenomyosis patients was only slightly lower (47.06% *vs* 64.42% P=0.028); however, their live birth rate was obviously declined (31.37% *vs* 54.815 P=0.004). One possible explanation for this is that after pregnancy, the size of the uterus of adenomyosis patients recovers to or even exceeds the level before pituitary function downregulation, which is deleterious for the maintenance of pregnancy (22). This reminds us that live birth rate is more appropriate than clinical pregnancy rate when choosing primary outcome in such studies of adenomyosis patients. Some studies have shown that for adenomyosis patients, it is better for us to freeze all embryos in fresh cycles, then perform frozen thawed embryo transfers in an estrogen-progesterone protocol after pretreatment with long-acting GnRH-a to fully shrink the size of the uterus (8).

However, the matched case-control study still has its limitation owing to its retrospective features even after controlling for age and other factors influencing the pregnancy outcome of IVF. First, in the adenomyosis group, though we completely excluded patients with endometrioma, we could not exclude patients with pelvic endometriosis because most adenomyosis patients did not receive laparoscopic surgery. In addition, adenomyosis in our study was diagnosed by transvaginal ultrasound (TVS) examination, which has limited specificity. Other studies have showed that the sensitivity of MRI is higher than that of ultrasonic examination in the diagnosis of adenomyosis, so there may be some misdiagnosis in our study (23). Future studies should include the size of the uterus which was indeed an important factor influencing IVF outcomes (19).

Taken together, the current matched case control study showed that even in the super long protocol with long-acting GnRH-a pretreatment for several cycles, adenomyosis still has adverse influences on the pregnancy outcome of IVF.

## Data Availability Statement

The raw data supporting the conclusions of this article will be made available by the authors, without undue reservation.

## Ethics Statement

This study has been approved by the Institutional Review Board (IRB) of First Affiliated Hospital of Zhengzhou University in January 2020. The patients/participants provided their written informed consent to participate in this study.

## Author Contributions

ZJ and BZ contributed to the conception, design, acquisition and interpretation of data, and drafting of the manuscript. SY and HL supervised the study. All authors contributed to the article and approved the submitted version.

## Funding

This study was supported by the Natural Science Foundation of China (NO:81801448).

## Conflict of Interest

The authors declare that the research was conducted in the absence of any commercial or financial relationships that could be construed as a potential conflict of interest.

## Publisher’s Note

All claims expressed in this article are solely those of the authors and do not necessarily represent those of their affiliated organizations, or those of the publisher, the editors and the reviewers. Any product that may be evaluated in this article, or claim that may be made by its manufacturer, is not guaranteed or endorsed by the publisher.
